# The impairment of induction chemotherapy for stage II nasopharyngeal carcinoma treated with intensity‐modulated radiotherapy with or without concurrent chemotherapy: A propensity score‐matched analysis

**DOI:** 10.1002/cam4.5199

**Published:** 2022-09-17

**Authors:** YuLin Lai, ChengTao Wang, XingLi Yang, ShaSha He, Yan Wang, Yong Chen

**Affiliations:** ^1^ Department of Radiation Oncology, The First Affiliated Hospital Sun Yat‐sen University Guangzhou China; ^2^ Department of Radiation Oncology, Sun Yat‐sen University Cancer Center, State Key Laboratory of Oncology in South China Collaborative Innovation Center for Cancer Medicine, Guangdong Key Laboratory of Nasopharyngeal Carcinoma Diagnosis and Therapy Guangzhou People's Republic of China

**Keywords:** concurrent chemoradiotherapy, induction chemotherapy, intensity‐modulated radiotherapy, nasopharyngeal carcinoma, propensity score‐matched analysis

## Abstract

**Objectives:**

To explore the efficacy of induction chemotherapy (IC) plus concurrent chemoradiotherapy (CCRT) in stage II nasopharyngeal carcinoma (NPC) treated with intensity‐modulated radiotherapy (IMRT).

**Methods:**

Totally, 450 eligible patients with staged II NPC on the basis of the 8th edition of the AJCC/UICC TNM staging system were eventually included from January 2010 to September 2020. The one‐to‐one propensity score‐matched (1:1 PSM) analysis was employed to balance variables. We conducted univariate and multivariate analysis of survival to identify prognostic factors and demonstrated the findings in the matching cohort.

**Results:**

In total, 141 pairs were selected by 1:1 PSM. IC + CCRT group in the matched data decreased 5‐year progression‐free survival (PFS, 75.5% vs. 88.0%, *p* = 0.032) and distant metastasis‐free survival (DMFS, 86.0% vs. 96.5%, *p* = 0.009). There was no significant difference in 5‐year overall survival (OS, 93.8% vs. 95.6%, *p* = 0.192) and locoregional relapse‐free survival (LRRFS, 87.1% vs. 94.3%, *p* = 0.169) compared with RT/CCRT. Multivariate analysis indicated that IC + CCRT was associated with significantly poor PFS (*p* = 0.024) and DMFS (*p* = 0.010). High neutrophil‐to‐lymphocyte ratio (>4.1) was negatively associated with OS (*p* = 0.034), PFS (*p* = 0.017) and DMFS (*p* = 0.001).

**Conclusion:**

Adding IC to CCRT or IMRT alone has decreased PFS and DMFS, therefore, IC should not be recommended in stage II NPC patients. No significant differences in OS and LRRFS were observed in stage II disease.

## 1 INTRODUCTION

Nasopharyngeal carcinoma (NPC) was a rare malignant tumor derived from nasopharyngeal epithelium, which was mainly endemic in southern China and Southeast Asia. According to the global cancer data in 2018, approximately 130,000 new cases of NPC, accounting for 0.7% of all new cases, and more than 72,000 deaths, accounting for 0.8% of all deaths.^1^, ^2^, ^3^ Radiotherapy (RT) alone or combined with chemotherapy was the main treatment modality for NPC on account of its unique anatomical location and sensitivity to RT.

The management for stage II NPC was controversial, and the effect of induction chemotherapy was unclear. A PSM analysis revealed that induction chemotherapy (IC) + RT significantly improved 5‐year overall survival (OS) and distant metastasis‐free survival (DMFS) of stage II NPC, compared with concurrent chemoradiothrapy (CCRT).^4^ Further subgroup analysis of two phase III trials also found that the addition of IC significantly improved 5 years OS and DMFS for T1‐T2N0‐N1 NPC patients.^5^ However, some studies have shown the different results. A retrospective research uncovered the addition of neoadjuvant chemotherapy to intensity‐modulated radiotherapy (IMRT) did not achieve survival benefit.^6^ Furthermore, adding IC to CCRT exerted an adverse effect on patients with stage II disease, significantly impaired 5‐year disease‐free survival (DFS) (80.6% vs. 88.5%, *p* = 0.043); nevertheless, similar outcome occurred in 5‐year OS (90.5% vs. 95.0%, *p* = 0.375).^7^ Similarly, another study published obtained similar results and revealed that IC caused deleterious effect for stage II disease, significantly decreased 5 years OS, progression‐free survival (PFS), locoregional failure‐free survival (LRFFS), and DMFS.^8^

As described above, till now, there was no consensus on the efficacy of IC in stage II NPC. It indicated the heterogeneity of stage II NPC. Except for N stage, there were some potential prognosis factors such as nutrition and inflammatory markers is not taken into account. Inflammatory markers based on several leukocytes, such as the neutrophil‐lymphocyte ratio (NLR) and the systemic immune‐inflammation index (SII), have been studied in NPC, and have been considered to be independent prognostic factors.^9^, ^10^, ^11^, ^12^ Cancer patients with systematic inflammation had worse prognosis than those without systematic inflammation.^13^, ^14^, ^15^

Nutritional status, in particular body mass index (BMI), was another aspect affecting the patients' prognosis and treatment responses.^16^ Numerous studies have shown pretreatment nutritional status was relevant to treatment response and clinical prognosis in a various of cancers, including NPC.^17^, ^18^, ^19^ Malnourished patients suffered more toxic reactions and therapy interruptions, leading to poorer outcomes.

Although there were some studies on the effect of IC in stage II NPC, the results were inconsistent, and the effects of systemic inflammation and nutritional status on prognosis were not considered in the studies. Therefore, we conducted a retrospective study incorporating systemic inflammation with nutritional status indicators to assess the effect of IC for stage II NPC in the IMRT era.

## 2 MATERIALS AND METHODS

### 2.1 Patient selection

A total of 450 eligibility patients with staged II NPC based on the eighth edition of the AJCC/UICC TNM staging system were retrospectively enrolled from January 2010 to September 2020. The inclusion criteria were as follows: (1) not less than 18 years old; (2) pathologically confirmed NPC; (3) stage II disease according to the 8th edition AJCC/UICC staging system; and (4) detailed pretreatment clinical information, hematological and biochemical data. Exclusion criteria were as follows: (1) less than 18 years old; (2) lactating or pregnant patients; and (3) lacking of pretreatment clinical information and laboratory data. The Research Ethics Committee of FAH‐SYSU reviewed and approved this study.

### 2.2 Pretreatment examination

All patients were assessed routinely with a comprehensive examination before treatment, including physical examination, fiber‐optic nasal endoscopy, magnetic resonance imaging (MRI) or contrast‐enhanced computed tomography of head and neck, chest radiography or enhanced computed tomography, abdominal ultrasonography or enhanced computed tomography, bone scan, or/and whole body positron emission tomography/computed tomography (PET/CT), electrocardiography, hematologic and biochemical tests.

### 2.3 Radiotherapy

In this study, all patients were treated with IMRT. The delineation and prescribed doses of gross target volume were in accordance with International consensus and guidelines.^20^ Gross tumor volume (GTV) included the primary nasopharynx tumor (GTVnx) and clinically metastatic lymph nodes (GTVnd), especially, enlarged retropharyngeal nodes were included in GTVnx as well due to adjacent to primary nasopharynx tumor. Clinical target volume (CTV) consisted of two parts called as high‐risk clinical target volume (CTV1) and low‐risk clinical target volume (CTV2). CTV1 was defined as the area where the nasopharyngeal GTV expanded 5–10 mm margin, whereas it only expanded 2–3 mm posteriorly near the brainstem and spinal cord to cover the high‐risk areas microscopic extension and the entire nasopharynx. CTV2 was defined as the area where CTV1 expanded 5–10 mm margin, whereas it only expanded 2–3 mm posteriorly near the brainstem and spinal cord to cover the low‐risk areas of microscopic extension. The prescribed doses to the planning target volumes of GTVnx, GTVnd, CTV1, and CTV2 were 68–70 Gy, 62–68 Gy, and 60 Gy, 54 Gy. Conventional schedule with once a day and 5 days every week was administered and RT was started on the first day of the first CCRT cycle.

### 2.4 Chemotherapy

IC regimens were as follows: triple combination of docetaxel (60 mg/m^2^, d1), cisplatin (60 mg/m^2^, d1) plus 5‐florouracil (600 mg/m^2^, d1–d5) (TPF), docetaxel (75 mg/m^2^, d1) plus cisplatin (75 mg/m^2^, d1) (TP), cisplatin (80 mg/m^2^, d1) plus 5‐florouracil (800 mg/m^2^, d1–d5)(PF)and gemcitabine (1 g/m^2^, d1, d8) plus cisplatin (80 mg/m^2^, d1) (GP), which were administered every 3 weeks. Concurrent chemotherapy (CCT) regimens mainly included cisplatin and nedaplatin every 3 weeks. Details of chemotherapy were provided in Table S4 and S5.

### 2.5 Clinical outcome and follow‐up

During the follow‐up period after treatment, the follow‐up was mainly carried out by telephone and clinic outpatient. Follow‐up frequency was usually every 3 months for the first 3 years, every 6 months for the next 2 years, and once a year after 5 years until death. Location and timing of tumor recurrence and metastasis were documented. Primary endpoint was PFS (the interval from pathological diagnosis time to date of death from any cause or the first presence of treatment failure including local recurrence and/or distant metastasis), and the secondary endpoints included OS (the interval from pathological diagnosis time to death date from any cause), DMFS (the interval from pathological diagnosis time to the first presence of distant metastasis lesion) and LRRFS (the interval from pathological diagnosis time to the first recurrence in the cervical and/or nasopharyngeal region after RT).

### 2.6 Statistical analysis

1:1 PSM analysis was computed without replacement using the nearest‐neighbor method with a match tolerance of 0.01. X‐tile software was used to establish optimal cut‐off value to convert continuous variables into categorical variables, including NLR and SII.^21^, ^22^ The Chi‐square test or Fisher's exact test was used to compare categorical variables between two groups. The cut‐off value of age was based on the median age. The Kaplan–Meier method and the log‐rank test were applied to estimate and compare survival outcomes. Multivariate Cox regression analysis was performed to estimate relation between variables with *p* ≤ 0.05 in the univariate analysis and OS, PFS, DMFS and LRRFS adopting a backward stepwise algorithm. Hazard ratio (HR) and 95% confidence interval (CI) were calculated by Cox proportional hazards model. SPSS version 25.0 was used to conduct PSM, univariate and multivariate analysis. GraphPad Prism 9 was applied for drawing Kaplan–Meier survival curves. A two‐sided *p* value < 0.05 was considered statistically significant difference.

## 3 RESULTS

### 3.1 Baseline characteristics

The clinical data of 450 restaged II NPC patients were selected and retrospectively reviewed. In the original data, some baseline characteristics were significantly distinct, including N category and SII, which *p* value was 0.002, 0.018, respectively (Table S1). We conducted univariate analysis to identify independent prognostic variables selected as matching variables during PSM (Table S2). From the original data, 141 pairs were selected by one‐to‐one PSM. The baseline characteristics of the two cohorts in the matched data were well‐balanced through PSM with all *p* values above 0.120 (Table 1). The median follow‐up time of IC + CCRT and RT/CCRT groups was 72.1 months (IQR = 20.1–107.1 months) and 60.5 months (IQR = 20.2–100.6 months), respectively. Furthermore, the treatment plans were also displayed (Table S4). After 1:1 match, the CCT regimens and cycles were similar in the two treatment arms (Table S5).

**TABLE 1:** Baseline characteristics in the matched data set

Characteristic	IC + CCRT (*N* = 141)	RT/CCRT (*N* = 141)	*p‐*value
Age, years			0.122
≤45	77 (54.6%)	64 (45.4%)	
>45	64 (45.4%)	77 (54.6%)	
Gender			0.787
Male	103 (73.0%)	105 (74.5%)	
Female	38 (27.0%)	36 (25.5%)	
BMI, kg/㎡			0.816
≤24	85 (60.3%)	84 (59.6%)	
>24	56 (39.7%)	57 (40.4%)	
T category (8th edition)			1.000
T1	46 (32.6%)	46 (32.6%)	
T2	95 (67.4%)	95 (67.4%)	
N category (8th edition)			0.776
N0	7 (5.0%)	6 (4.3%)	
N1	134 (95.0%)	135 (95.7%)	
NLR			1.000
≤4.1	128 (90.8%)	128 (90.8%)	
>4.1	13 (9.2%)	13 (9.2%)	
SII			0.861
≤1122.1	127 (90.1%)	128 (90.8%)	
>1122.1	14 (9.9%)	13 (9.2%)	

Abbreviations: BMI, body mass index; CCRT, concurrent chemoradiotherapy; IC, induction chemotherapy; NLR, the neutrophil‐lymphocyte ratio; RT, radiotherapy; SII, the systemic immune‐inflammation index.

### 3.2 Survival outcomes

**FIGURE 1:**
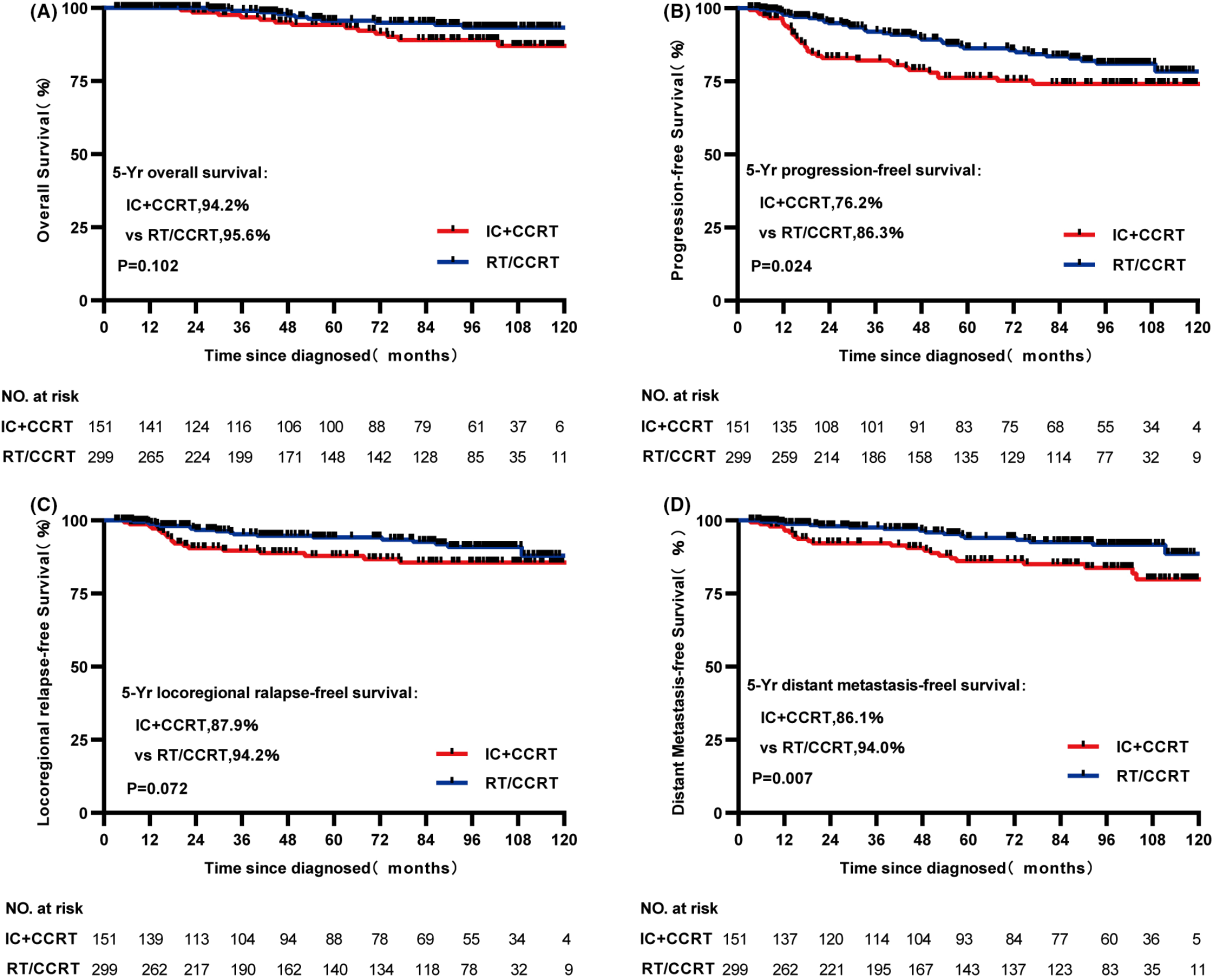
Kaplan–Meier survival curves of OS (A), PFS (B), LRRFS (C) and DMFS (D) for patients with stage II NPC receiving IC + CCRT or RT/CCRT treatment in the original data set. DMFS, distant metastasis‐free survival; LRRFS, locoregional recurrence‐free survival; OS, overall survival; PFS, progression‐free survival.

**FIGURE 2:**
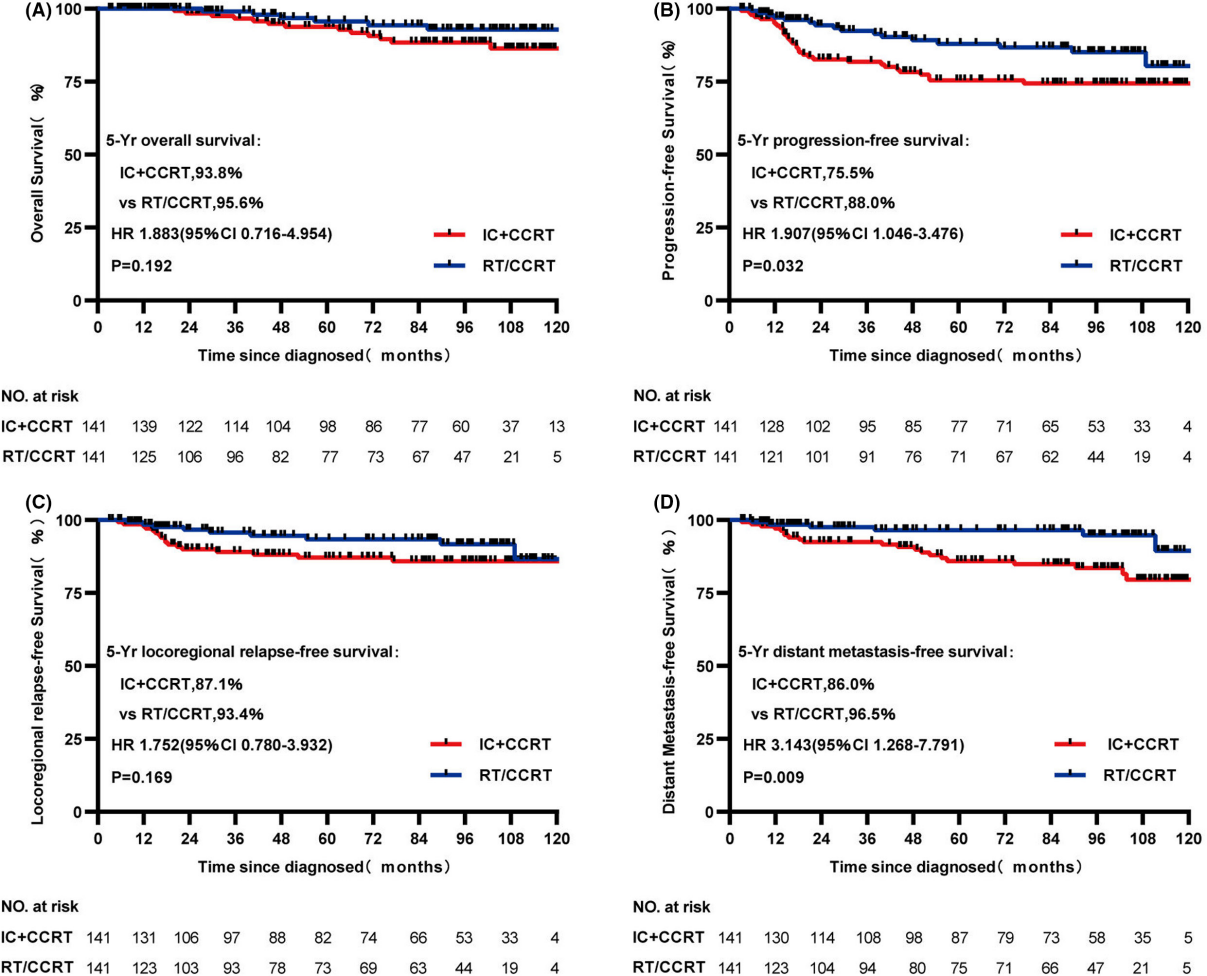
Kaplan–Meier survival curves of OS (A), PFS (B), LRRFS (C), and DMFS (D) for patients with stage II NPC receiving IC + CCRT or RT/CCRT treatment in the match data set. DMFS, distant metastasis‐free survival; LRRFS, locoregional recurrence‐free survival; OS, overall survival; PFS, progression‐free survival.

From the original data, although statistically significant difference in OS and LRRFS was not found between IC + CCRT and RT/CCRT groups (both *p* > 0.05, 5‐year OS: 94.2% vs. 95.6%, respectively, *p* = 0.102, Figure 1A; 5‐year LRRFS: 87.9% vs. 94.2%, respectively, *p* = 0.072, Figure 1C), PFS and DMFS were significant statistically between two treatment cohorts (both *p* < 0.05, 5‐year PFS: 76.2% vs. 86.3%, respectively, *p* = 0.024, Figure 1B; 5‐year DMFS: 86.1% vs. 94.0%, respectively, *p* = 0.007, Figure 1D). In the matched data set, survival outcomes were similar to that before match. 5‐year PFS and DMFS in the IC + CCRT cohort were significantly worse and statistically significant (both *p* < 0.05, 5‐year PFS: 75.5% vs. 88.0%, respectively, *p* = 0.032, Figure 2B; 5‐year DMFS: 86.0% vs. 96.5%, respectively, *p* = 0.009, Figure 2D), but not statistically significant in OS and LRRFS (both *p* > 0.05, 5‐year OS: 93.8% vs. 95.6%, respectively, *p* = 0.192, Figure 2A; 5‐year LRRFS: 87.1% vs. 94.3%, respectively, *p* = 0.169, Figure 2C) compared with the RT/CCRT cohort.

### 3.3 Univariate and multivariate analysis

We performed univariate and multivariate analysis for demographic and clinicopathologic variables to identify prognostic factors. Consistent with univariate analysis (Table 2), age, NLR and treatment modality were statistically significant (all *p* < 0.05) in multivariate Cox analysis (Table S3). Younger patients (≤45) were associated with better LRRFS (HR = 0.402, 95% CI = 0.169–0.957, *p* = 0.033). High NLR (>4.1) achieved worse survival outcomes of 5‐year OS (HR = 3.509, 95% CI = 1.017–12.104, *p* = 0.034), 5‐year PFS (HR = 2.653, 95% CI = 1.193–5.898, *p* = 0.017), 5‐year DMFS (HR = 4.762, 95% CI = 1.914–11.848, *p* = 0.001). In term of the treatment modality, the effect of IC on survival outcomes remained consistent with univariate analysis after taking into account prognostic factors. IC + CCRT arm decreased survival rate of 5‐year PFS (HR = 2.009, 95% CI = 1.097–3.681, *p* = 0.024) and 5‐year DMFS (HR = 3.324, 95% CI = 1.335–8.281, *p* = 0.010).

**TABLE 2:** Univariate analysis in matched data set

Variable	OS		PFS		DMFS		LRRFS	
	HR (95% CI)	*p*‐value	HR (95% CI)	*p*‐value	HR (95% CI)	*p*‐value	HR (95% CI)	*p*‐value
Age (years)								
>45 vs. ≤45	1.170 (0.474–2.884)	0.733	0.702 (0.393–1.252)	0.228	0.604 (0.271–1.347)	0.213	0.402 (0.169–0.957)	0.033
Gender								
Female vs. male	1.329 (0.505–3.497)	0.563	1.346 (0.731–2.479)	0.338	1.437 (0.645–3.198)	0.372	1.325 (0.576–3.048)	0.506
BMI (kg/m^2^)								
≥24 vs. <24	0.581 (0.221–1.529)	0.265	0.476 (0.252–0.900)	0.019	0.297 (0.112–0.785)	0.009	0.482 (0.203–1.148)	0.092
T category (8th edition)								
T2 vs. T1	1.413 (0.537–3.720)	0.481	1.289 (0.700–2.376)	0.414	2.036 (0.821–5.050)	0.117	1.594 (0.669–3.796)	0.288
N category (8th edition)								
N1 vs. N0	0.864 (0.115–6.474)	0.887	0.685 (0.213–2.206)	0.524	1.225 (0.166–9.036)	0.842	0.335 (0.100–1.118)	0.062
NLR								
>4.1 vs. ≤4.1	3.509 (1.017–12.104)	0.034	2.986 (1.391–6.408)	0.003	5.829 (2.427–13.997)	0.000	1.750 (0.523–5.861)	0.358
SII								
>1122.1 vs. ≤1122.1	2.240 (0.517–9.700)	0.268	2.445 (1.093–5.470)	0.025	4.147 (1.664–10.338)	0.001	1.746 (0.522–5.842)	0.360
Treatment modality								
IC + CCRT vs. RT/CCRT	1.883 (0.716–4.954)	0.192	1.907 (1.046–3.476)	0.032	3.143 (1.268–7.791)	0.009	1.752 (0.780–3.932)	0.169

Abbreviations: BMI, body mass index; CCRT, concurrent chemoradiotherapy; CI, confidence interval; DMFS, distant metastasis‐free survival; HR, hazard ratio; IC, induction chemotherapy; LRRFS, locoregional recurrence‐free survival; NLR, the neutrophil‐lymphocyte ratio; OS, overall survival; PFS, progression‐free survival; RT, radiotherapy; SII, the systemic immune‐inflammation index.

## 4 DISCUSSION

In the present study, we demonstrated that patients receiving IC + CCRT achieved unfavorable survival outcomes, although no statistical difference was observed in OS and LRRFS, as compared to RT/CCRT. By means of the application of PSM, this study balanced the difference and provided a fair comparison in matched patients, therefore, the conclusion should be reliable.

NPC cells were sensitive to RT. For stage I NPC, RT alone was sufficient, and the 5‐year survival rate could reach more than 96%.^23^ However, RT alone might not be sufficient for stage II disease. The latest NCCN guideline recommended CCRT + ‐ACT/IC for stage II NPC,^24^ therefore, Platinum‐based CCT was often used in clinical work to improve the outcome of stage II patients. Because the platinum drugs targeted DNA by forming intra‐stranded and inter‐stranded crosslinking and produced more DNA damage to increased sensitivity to radiation.^25^

To further improve the survival rate, one important strategy was IC followed by CCRT. In 2018, a PSM analysis found that IC + RT significantly improved 5‐year OS and DMFS for stage II NPC, compared with CCRT arm.^4^ Notably, more patients completed IMRT within <44 days in IC + RT arm. Another subgroup analysis of two phase III trials yielded similar results.^5^ But most patients classified as stage II NPC were stage IIB with poor prognosis attributed to the relatively high incidence of distant metastases and patients were rarely classified as stage IIA. Therefore, local RT alone was not sufficient and needed to add IC to RT to reduce the incidence of distant metastasis.

Whereas a retrospective study, which enrolled 242 patients treated with IMRT in Zhejiang Cancer Hospital, found that 5‐year survival rates, including LRRFS, DMFS, PFS, and OS, were approximate among the IMRT alone, IC + IMRT, IC + CCRT, and CCRT groups.^6^ Notably, there was obvious difference in the number of patients who received CCRT and IC + CCRT (25 vs. 132 patients), although other characteristics were well‐balanced between four cohorts.

Reversely, in 2020, a retrospective study including 445 patients in two hospitals of southern China was carried out and showed that IC plus CCRT significantly decreased 5‐year DFS (80.6% vs. 88.5%, *p* = 0.043); however, no statistically significant difference was observed in 5‐year OS (90.5% vs. 95.0%, *p* = 0.375).^**7**^ In addition, radiation technique, N category and subgroup were not well balanced (*p* = 0.034, <0.001, <0.001, respectively). IC + CCRT arm had more N1 diseases and stage T2N1M0 compared with CCRT. Some studies had found that patients with N1 disease and stage T2N1M0 had worse prognosis than those with N0 disease and other groups (T1N1M0 and T2N0M0) because of the high incidence of distant metastasis.^26^, ^27^, ^28^, ^29^, ^30^ Another study yielded similar results and reported that IC caused deleterious effect for stage II disease, significantly decreased 5‐year OS, PFS, LRFFS and DMFS.^8^ The potential reason was that baseline data such as median age, stage T2 and completion of CCT were uneven between IC + CCRT and CCRT arm. Obviously, both cohorts had small sample sizes. Moreover, IC + CCRT arm had more advanced stage (T2), higher proportion of older patients and higher proportion of patients who had completed two or three cycles of CCT. Patients with T1 or received 2–3 cycles of CCT achieved better outcomes than those with T2 or received 1 cycle of CCT.^27^, ^28^, ^31^, ^32^ Furthermore, older patients had poor prognosis.^33^

We thought there might be two important reasons for interpreting the conflicting results in these studies. Firstly, the imbalance of the two baseline data sets described above, such as sample size, stage T, stage N and degree of CCT completion, which were associated with clinical prognosis. Secondly, some potential prognostic factors such as nutrition and/or inflammatory status were ignored.

Inflammatory markers including the NLR and SII, have been verified in NPC.^9^, ^10^, ^11^, ^12^ Neutrophil was an inflammatory cell that promoted tumor development by secreting several factors such as hepatocyte growth factor, transforming growth factor‐β, and vascular endothelial growth factor.^34^, ^35^, ^36^ Platelet was an important component of thrombosis process and was involved in tumor growth, dissemination, and angiogenesis.^37^ While lymphocytes were involved in immune monitoring to eliminate cancer cells,^38^ hence, NLR and SII both reflected the relative ability to promote and kill tumors. Nutritional status, in particular BMI, was another important prognostic factor. In comparison with low‐BMI patients, high‐BMI patients achieved favorable clinical outcomes. Malnutrition impaired immune status and reduced patient's ability to tolerate anti‐tumor therapies, including surgery, RT, and chemotherapy.^39^, ^40^

In the present study, we not only considered the clinical demographic characteristics such as age, gender and TNM staging affecting the prognosis but also included in the systemic inflammation indexes (such as NLR and SII) and nutrition indicator (such as BMI). The statistics method of 1:1 PSM was conducted to balance the baseline data between two arms, reduce the bias and ensure the data comparable in both groups by simulating prospective studies. The matched data showed that all prognostic indicators were balanced between the two groups, both in clinical demography and chemotherapy regimens and duration. Secondly, the relatively large sample sizes and long follow‐up duration increased the reliability of the data and conclusion made.

Yet there were several limitations worth considering in our study. Firstly, it was a still retrospective study and inevitably existed bias, although the statistical method of PSM was used. Secondly, the study lacked of chemotherapy and radiation‐related adverse events, such as radiation‐related mucositis, chemotherapy‐related bone marrow suppression, and liver and kidney injury. Moreover, pre‐treatment plasma Epstein–Barr virus DNA (EBV DNA) as a critical prognostic biomarker was not included.^3^, ^41^ Due to the inconsistency of quantitative EBV DNA test kits in our hospital leading to different reference standards, it was not included. It is hoped that the standardization of EBV DNA quantitative detection can be solved in the future.

## 5 CONCLUSION

In conclusion, the addition of IC did not improve the overall survival and locoregional recurrence‐free survival of patients with stage‐II NPC, on the contrary, it significantly decreased PFS and DMFS, when compared with IMRT alone or CCRT. Therefore, in the future, well‐designed phase 3, multicenter, prospective, randomized, controlled trials are needed to further verify whether adding IC to CCRT or IMRT alone should be recommended for stage II NPC.

## AUTHOR CONTRIBUTIONS

**YuLin Lai:** Data curation (lead); formal analysis (lead); investigation (lead); software (lead); visualization (lead); writing – original draft (lead). **Chengtao Wang:** Data curation (lead); formal analysis (lead); supervision (equal); validation (lead); writing – original draft (equal). **Xing‐Li Yang:** Data curation (lead); formal analysis (equal); software (lead); visualization (equal). **ShaSha He:** Funding acquisition (lead); methodology (lead); software (lead); writing – review and editing (lead). **yan wang:** Conceptualization (lead); methodology (lead); supervision (lead); writing – review and editing (lead). **yong chen:** Conceptualization (lead); methodology (lead); resources (lead); supervision (lead); writing – review and editing (lead).

## CONFLICTS OF INTEREST

All authors declare that they have no known financial interests or personal interests in this paper.

## ETHICS STATEMENT

The Research Ethics Committee of FAH‐SYSU reviewed and approved this study.

## Supporting information


Table S1
Table S2
Table S3
Table S4
Table S5

## Data Availability

Research data were not shared.
