# Supplementary material for: The impairment of induction chemotherapy for stage II nasopharyngeal carcinoma treated with intensity‐modulated radiotherapy with or without concurrent chemotherapy: A propensity score‐matched analysis
Source: Cancer Med. 2022 Sep 17;12(3):2970–8. doi: 10.1002/cam4.5199 (PMC9939148; doi:10.1002/cam4.5199)
Supplement: Supplementary file 1 — Table S1 Table S2 Table S3 Table S4 Table S5 [file CAM4-12-2970-s001.docx]

**Supplementary Table S1. Baseline characteristics and treatment details of the patients in the original data set.**

| Characteristic | IC+CCRT (N=151) | RT/CCRT (N=299) | *P*-value |
| --- | --- | --- | --- |
| **Age, year** |  |  | 0.089 |
| ≤45 | 81 (53.6%) | 135 (45.2%) |  |
| >45 | 70 (46.4%) | 164 (54.8%) |  |
| **Gender** |  |  | 0.632 |
| Male | 111 (73.5%) | 226 (75.6%) |  |
| Female | 40 (26.5%) | 73 (24.4%) |  |
| **BMI, kg/㎡** |  |  | 0.489 |
| ≤24 | 88 (58.3%) | 164 (54.8%) |  |
| >24 | 63 (41.7%) | 135 (45.2%) |  |
| **T Stage (8th edition)** |  |  | 0.125 |
| T1 | 52 (34.4%) | 82 (27.1%) |  |
| T2 | 99 (65.6%) | 217 (72.6%) |  |
| **N Stage (8th edition)** |  |  | 0.002 |
| N0 | 7 (4.6%) | 43 (14.4%) |  |
| N1 | 144 (95.4%) | 256 (85.6%) |  |
| **NLR** |  |  | 0.292 |
| ≤4.1 | 132 (87.4%) | 271 (90.6%) |  |
| >4.1 | 19 (12.6%) | 28 (9.4%) |  |
| **SII** |  |  | 0.018 |
| ≤1122.1 | 128 (84.8%) | 275 (92.0%) |  |
| >1122.1 | 23 (15.2%) | 24 (8.0%) |  |

Abbreviations: IC = induction chemotherapy; CCRT = concurrent chemoradiotherapy; RT = radiotherapy; BMI = body mass index; NLR = the neutrophil‐lymphocyte ratio; SII = the systemic immune‐inflammation index.

**Supplementary Table S2. Univariate analysis of the effect of prognostic factors in original data set.**

| Variable | OS | | PFS | | DMFS | | LRRFS | |
| --- | --- | --- | --- | --- | --- | --- | --- | --- |
|  | HR (95% CI) | *P*-value | HR (95% CI) | *P*-value | HR (95% CI) | *P*-value | HR (95% CI) | *P*-value |
| **Age at diagnosis (years)** |  |  |  |  |  |  |  |  |
| >45 vs ≤45 | 1.367 (0.612-3.056) | 0.444 | 0.775 (0.485-1.239) | 0.286 | 0.709 (0.369-1.360) | 0.298 | 0.444 (0.219-0.904) | 0.021 |
| **Gender** |  |  |  |  |  |  |  |  |
| Female vs Male | 1.018 (0.404-2.565) | 0.970 | 1.134 (0.670-1.918) | 0.639 | 1.245 (0.617-2.510) | 0.540 | 1.387 (0.682-2.819) | 0.364 |
| **BMI (kg/m2)** |  |  |  |  |  |  |  |  |
| ≥24 vs <24 | 1.005 (0.450-2.243) | 0.991 | 0.584 (0.358-0.957) | 0.031 | 0.425 (0.206-0.875) | 0.017 | 0.453 (0.218-0.940) | 0.029 |
| **T category (8th edition)** |  |  |  |  |  |  |  |  |
| T2 vs T1 | 1.243 (0.532-2.906) | 0.615 | 1.073 (0.655-1.758) | 0.778 | 1.058 (0.540-2.072) | 0.869 | 1.431 (0.688-2.973) | 0.335 |
| **N category (8th edition)** |  |  |  |  |  |  |  |  |
| N1 vs N0 | 2.563 (0.346-18.990) | 0.339 | 1.595 (0.642-3.960) | 0.310 | 4.260 (0.584-31.077) | 0.119 | 0.723 (0.281-1.862) | 0.500 |
| **NLR** |  |  |  |  |  |  |  |  |
| >4.1 vs ≤4.1 | 1.822 (0.543-6.115) | 0.324 | 2.306 (1.237-4.298) | 0.007 | 3.653 (1.721-7.756) | 0.000 | 2.159 (0.896-5.206) | 0.079 |
| **SII** |  |  |  |  |  |  |  |  |
| >1122.1 vs ≤1122.1 | 1.433 (0.337-6.101) | 0.624 | 2.115 (1.081-4.138) | 0.025 | 3.489 (1.593-7.640) | 0.001 | 1.986 (0.769-5.127) | 0.148 |
| **Treatment modality** |  |  |  |  |  |  |  |  |
| IC+CCRT vs RT/CCRT | 1.930 (0.864-4.310) | 0.102 | 1.699 (1.066-2.708) | 0.024 | 2.352 (1.234-4.484) | 0.007 | 1.806 (0.939-3.475) | 0.072 |

Abbreviations: OS = overall survival; PFS = progression-free survival; DMFS = distant metastasis-free survival; LRRFS = locoregional recurrence-free survival; HR = hazard ratio; CI = confidence interval; BMI = body mass index; NLR = the neutrophil‐lymphocyte ratio; SII = the systemic immune‐inflammation index; IC = induction chemotherapy; CCRT = concurrent chemoradiotherapy; RT = radiotherapy.

**Supplementary Table S3. Multivariate Cox analysis of 141 patients in matched data set.**

| Variable | OS | | PFS | | DMFS | | LRRFS | |
| --- | --- | --- | --- | --- | --- | --- | --- | --- |
|  | HR (95% CI) | *P*-value | HR (95% CI) | *P*-value | HR (95% CI) | *P*-value | HR (95% CI) | *P*-value |
| **Age (years)** |  |  |  |  |  |  |  |  |
| >45 vs ≤45 | - | - | - | - | - | - | 0.402 (0.169-0.957) | 0.033 |
| **NLR** |  |  |  |  |  |  |  |  |
| >4.1 vs ≤ 4.1 | 3.509 (1.017-12.104) | 0.034 | 2.653 (1.193-5.898) | 0.017 | 4.762 (1.914-11.848) | 0.001 | - | - |
| **Treatment modality** |  |  |  |  |  |  |  |  |
| IC+CCRT vs RT/CCRT | - | - | 2.009 (1.097-3.681) | 0.024 | 3.324 (1.335-8.281) | 0.010 | - | - |

Abbreviations: OS = overall survival; PFS = progression-free survival; DMFS = distant metastasis-free survival; LRRFS = locoregional recurrence-free survival; HR = hazard ratio; CI = confidence interval; NLR = the neutrophil‐lymphocyte ratio; IC = induction chemotherapy; CCRT = concurrent chemoradiotherapy; RT = radiotherapy.

**Supplementary Table S4. Chemotherapy regimen and cycle in the original data set.**

| Variable | IC+CCRT (N=151) | RT/CCRT (N=299) |
| --- | --- | --- |
| **IC regimen** |  |  |
| TPF | 54 (35.8%) | - |
| TP | 60 (39.7%) | - |
| PF | 32 (21.2%) | - |
| GP | 5 (3.3%) | - |
| **IC cycle** |  |  |
| ≤2 | 85 (56.3%) | - |
| >2 | 66 (43.7%) | - |
| **CCT regimen** |  |  |
| Not | 38 (25.2%) | 84 (28.1%) |
| DDP or NDP | 103 (68.2%) | 203 (67.9%) |
| Others | 10 (6.6%) | 12 (4.0%) |
| **CCT cycle** |  |  |
| 0 | 38 (25.2%) | 84 (28.1%) |
| 1 | 18 (11.9%) | 18 (6.0%) |
| 2 | 87 (57.6%) | 154 (51.5%) |
| 3 | 8 (5.3%) | 43 (14.4%) |

Abbreviations: IC = induction chemotherapy; CCRT = concurrent chemoradiotherapy; CRT = concurrent chemotherapy; RT = radiotherapy; TPF = docetaxel–cisplatin–5-florouracil; TP = docetaxel–cisplatin; PF = cisplatin–5-florouracil; GP = gemcitabine-cisplatin; DDP/P = cisplatin; NDP = nedaplatin.

**Supplementary Table S5. Chemotherapy regimen and cycle in the matched data set.**

| Variable | IC+CCRT (N=141） | RT/CCRT (N=141) |
| --- | --- | --- |
| **IC regimen** |  |  |
| TPF | 49 (34.8%) | - |
| TP | 56 (39.7%) | - |
| PF | 31 (22.0%) | - |
| GP | 5 (3.5%) | - |
| **IC cycle** |  |  |
| ≤2 | 81 (57.4%) | - |
| >2 | 60 (42.6%) | - |
| **CRT regimen** |  |  |
| Not | 36 (25.5%) | 36 (25.5%) |
| DDP+NDP | 95 (67.4%) | 98 (69.5%) |
| Others | 10 (7.1%) | 7 (5.0%) |
| **CRT cycle** |  |  |
| 0 | 36 (25.5%) | 36 (25.5%) |
| 1 | 17 (12.1%) | 11 (7.8%) |
| 2 | 81 (57.4%) | 70 (49.6%) |
| 3 | 7 (5.0%) | 24 (17.0%) |

Abbreviations: IC = induction chemotherapy; CCRT = concurrent chemoradiotherapy; CRT = concurrent chemotherapy; RT = radiotherapy; TPF = docetaxel–cisplatin–5-florouracil; TP = docetaxel–cisplatin; PF = cisplatin–5-florouracil; GP = gemcitabine-cisplatin; DDP/P = cisplatin; NDP = nedaplatin.
